# When the endothelium is on fire: bridging thromboinflammation and data-driven prognosis in pulmonary embolism

**DOI:** 10.1016/j.rpth.2026.106845

**Published:** 2026-07-13

**Authors:** Nikolaos Kotsiou, Paschalis Evangelidis

**Affiliations:** Second Propedeutic Department of Internal Medicine, Hippocration Hospital, Aristotle University of Thessaloniki, Thessaloniki, Greece

Pulmonary embolism (PE) constitutes one of the most common manifestations of venous thromboembolism, with substantial morbidity and mortality [1]. Specifically, based on a large epidemiological analysis of the World Health Organization mortality database from 2001 to 2023, while global age-standardized PE-related mortality decreased from 3.49 to 2.42 deaths per 100,000 population, PE continues to represent a major cause of cardiovascular mortality and disease burden [2].

Additionally, despite the fact that several advances have been made in this field, resulting in prompt diagnosis, effective and safe anticoagulant treatment, and implementation of multidisciplinary care, prediction of early clinical deterioration is considered a challenge. Notably, this is of paramount importance in patients who are initially classified outside the highest-risk groups based on standard classification scores but eventually experience rapid hemodynamic compromise or even death [3]. Indeed, current prognostic tools, such as the established PE Severity Index (PESI), have improved our everyday clinical decision-making [4]. Nevertheless, these scores include, mainly, variables associated with clinical severity, right ventricular strain, cardiac injury, and metabolic stress [3]. Therefore, the biological processes that precede or accompany clinical deterioration are not fully captured.

Thus, an important gap arises: PE is not only a syndrome characterized by mechanical obstruction of the pulmonary circulation but constitutes a thromboinflammatory syndrome in which endothelial activation, platelet recruitment, coagulation imbalance, impaired fibrinolysis, and innate immune activation interact [5]. Furthermore, the endothelium is not a passive bystander in this process [6]. Hypoxia, shear stress, inflammatory mediators, and damage-associated signals are detected and integrated by the endothelium, leading to a shift toward a proadhesive, procoagulant, and inflammatory state [6]. Therefore, endothelial injury may “act” as a biological amplifier of PE severity.

Building on this rationale, Siddiqui et al. [7], in their study published in the latest issue of Research and Practice in Thrombosis and Haemostasis, address these missing biological layers in PE risk evaluation. In a large cohort of 500 patients with a confirmed diagnosis of acute PE, the authors assessed a broad panel of biomarkers reflecting endothelial dysfunction, platelet activation, coagulation, fibrinolysis, systemic inflammation, and hematologic immune imbalance. Patients’ blood samples were collected within 24 to 72 hours following a PE diagnosis, and biomarkers were assessed in relation to 30-day mortality.

In their robust analysis, various findings were reported. First, patients who died within 30 days after a PE diagnosis exhibited higher levels of von Willebrand factor, P-selectin, tissue factor, tissue plasminogen activator, plasminogen activator inhibitor-1 (PAI-1), D-dimer, C-reactive protein, interleukin-6, tumor necrosis factor (TNF)-α, neutrophil-to-lymphocyte ratio (NLR), platelet-to-lymphocyte ratio, and systemic immune-inflammation index. On the contrary, factor (F)VII, FX, FXIII-A, and thrombin-activatable fibrinolysis inhibitor were lower in nonsurvivors. These differences may reflect the development of a biological state characterized by vascular injury, inflammatory activation, dysregulated coagulation, and a defective fibrinolytic balance in patients with PE who subsequently experience poor outcomes.

Moreover, the recursive partitioning analysis that was performed is particularly crucial for clinical interpretation [7]. NLR, PAI-1, and TNF-α emerged as key predictors of 30-day mortality [7], findings potentially consistent with the pathogenesis of acute PE. NLR is an easily calculated and widely available marker of inflammatory imbalance that has been shown to be a predictor of mortality in several other clinical entities, including hematological malignancies [8]. PAI-1 reflects impaired fibrinolysis and may identify patients in whom thrombus persistence and vascular obstruction are more pronounced [9]. In addition, TNF-α, as a proinflammatory cytokine, drives inflammatory activation, which may further promote vascular injury [10]. Therefore, the aforementioned markers suggest that early mortality in PE may be driven not only by clot size or right ventricular strain but also by thromboinflammation and impaired thrombus resolution.

The study has several strengths. First, a large cohort of PE patients with a broad biomarker panel was included. Furthermore, sample size calculation methods were adequate for the study design and the primary research question. Second, quantifiable thresholds were provided, offering potentially clinically meaningful findings. Third, easily measured biomarkers from different biological pathways were evaluated. Finally, the researchers highlighted the potential use of simple hematologic indices in the prediction of PE-associated mortality, particularly the NLR, which can be easily calculated even in resource-limited clinical settings. A useful prognostic biomarker should be easy to measure, reproducible, affordable, and informative beyond existing risk scores [11].

At the same time, several steps are needed before the integration of the above-mentioned biomarkers into everyday practice. Specifically, these findings arose from a single-center study, while prospective validation in larger, multicenter, and international cohorts is required. In addition, the timing of blood sample collection is another significant issue. Thromboinflammatory and endothelial injury markers are dynamic, and a single measurement may not capture the full trajectory of disease progression. Anticoagulation, reperfusion therapies, anti-inflammatory treatments, comorbidities, active cancer, infections, and chronic inflammatory states may also influence biomarker levels. These factors should be carefully taken into account in future similar studies.

Another challenge is the definition (and selection) of clinically useful cutoffs. The thresholds proposed in the study are promising, but they should be validated externally before being incorporated into decision algorithms. Moreover, it is important to determine whether these biomarkers improve existing, well-established clinical stratification models, such as PESI, simplified PESI, and right ventricular imaging-based stratification [12]. The key question is not whether the study’s biomarkers are statistically associated with mortality, but whether they can affect our clinical decisions, improving patient outcomes.

Collaboration with data scientists can be helpful for this objective. The increasing availability of clinical, laboratory, imaging, and outcome data creates an opportunity to build more precise prediction tools. Machine learning (ML) may help us to identify complex relationships between biomarkers and clinical outcomes [13,14]. In PE, ML models have already been explored for diagnosis, risk stratification, and prognosis [15]. External validation, calibration, interpretability, bias assessment, and cost-effectiveness analyses are essential in order to introduce these algorithms into clinical decision-making. Additionally, ML models, which will also incorporate mechanistic biomarkers such as NLR, PAI-1, and TNF-α may be more useful than “purely” data-driven models in the prediction of PE mortality and other poor outcomes.

The possible implications extend beyond PE. The relatively high frequency of venous thromboembolism allows large cohorts to be recruited for biomarker discovery and validation [16]. In contrast, rare thromboinflammatory disorders, such as thrombotic microangiopathies (TMAs), often lack the large datasets needed for robust biomarker discovery and validation. In TMAs, endothelial injury, complement dysregulation, platelet activation, and microvascular thrombosis are central to disease pathogenesis, but available cohorts are often small, with substantial heterogeneity across disease subtypes. Thus, multicenter collaboration is essential. The above-described strategy used in PE, integrating mechanistic biomarkers with data-driven predictive models, could inspire similar approaches in TMAs and other endothelial injury syndromes [17].

Finally, the next step should not be limited to the prediction of mortality. PE survivors may experience several long-term complications, such as persistent dyspnea, exercise limitation, anxiety, reduced quality of life, chronic thromboembolic disease, or chronic thromboembolic pulmonary hypertension [18]. Thus, patient-reported outcomes should be incorporated into future biomarker studies; prognostic biomarkers should not only predict death but also assist in identifying patients at risk of persistent symptoms, poor recovery, and long-term disability.

Summarizing, the work by Siddiqui et al. [7] offers an important bridge between translational research and prognostic data science in PE. Their findings support the concept that an interaction between thrombosis, inflammation, endothelial injury, and fibrinolytic failure contributes to early PE outcomes. Before the integration of these biomarkers into routine clinical practice, several questions remain unanswered. Prospective multicenter studies are needed to confirm generalizability, refine evidence-based cutoffs, evaluate longitudinal measurements, and define how these markers should be combined with current prognostic scores. Cost-effectiveness analyses are equally important, particularly if broad biomarker panels are proposed. For now, this study reminds us that in PE, the clot is only part of the story. The vascular response to the clot may be just as important.
